# Knockdown of long non-coding RNA H19 inhibits multiple myeloma cell growth via NF-κB pathway

**DOI:** 10.1038/s41598-017-18056-9

**Published:** 2017-12-22

**Authors:** Yuanyuan Sun, Jing Pan, Ning Zhang, Wei Wei, Shanshan Yu, Limei Ai

**Affiliations:** Department of Hematology, the First Affiliated Hospital of Jinzhou Medical University, Jinzhou, Liaoning China

## Abstract

Long non-coding RNAs (lncRNAs) are implicated in the complex network of cancer including Multiple myeloma (MM) and play important roles in tumor development. lncH19 was significantly up-regulated in multiple cancer types, suggesting it is a potential oncogene. However, the exact functions and downstream mechanisms are largely unknown. This study aimed to investigate whether H19 participates in the cell growth of MM and elucidate the underlying mechanism. We found that H19 was abnormally overexpressed in MM cell lines and sorted CD138^+^ MM bone marrow tissues. H19 knockdown induced by shRNA transfection significantly inhibited proliferation, viability and colony formation in MM cells, as well as inactivated NF-κB pathway. Moreover, combination treatment of H19 knockdown and NF-κB suppression (induced by specific inhibitor PDTC) produced synergistically inhibitory effects. Bone marrow expression of H19 was positively associated with circulating IL-6 or IL-8 level in the same MM patients. And patients with high expression of H19 had a lower survival rate. Taken together, we confirmed the abnormal upregulation of a novel lncRNA, H19, in human MM. H19 was involved in MM cell growth. The linkage between H19 and NF-κB pathway may provide a novel interpretation for the mechanism of H19’s growth regulation in MM.

## Introduction

Multiple myeloma (MM) is the second most common hematological cancer in the USA, and the 5-year survival rate reported has increased from 26.5% in 1975 to 44.9% in 2010 due to available newer and more effective treatment options^1,2^. However, although improvement on survival has been made, the treatment responses are transient. The recent availability of several novel drugs with different and innovative mechanisms of action has increased the therapeutic options, for example daratumumab, elotuzumab, carfilzomib, ixazomib, and panobinostat, but complexity in patient management was also increased. MM is still not considered curable, and the molecular mechanisms involved in its development are not well characterized^3,4^.

Long non-coding RNAs (lncRNAs) are a newly discovered type of non-coding RNAs, which are greater than 200 nucleotides in length and not translate into a protein. They are involved in many biological processes, such as cellular differentiation, cell growth, cell cycle regulation as well as cancer progression and metastasis^5,6^. Although they are not that well characterized as small non-coding miRNAs, there have been many studies showing that lncRNAs are abnormally expressed in human cancers, and could play a critical role in tumorigenesis. Depending on the cellular contexts and gene expressions they regulate, lncRNAs may function as tumor suppressors or oncogenes. For example, as one of the first identified lncRNAs, HOTAIR is highly expressed in primary breast tumors and metastases, and its expression is a powerful prognosis marker of patient outcomes such as metastasis and survival^7^. Otherwise, Loc285194 is a direct target of p53 in colorectal cancer. It is downregulated in colon tumor specimens, suppresses cell growth, and functions as a tumor suppressor^8^.

Among the identified functional lncRNAs, H19 is the first one discovered in 11p15.5, H19/IGF2 locus^9^. It is highly expressed in fetus but begins to silence after birth except in few tissues like mammary gland, adrenal gland and uterus^10^. Mutation of H19 in mouse zygotes causes prenatal lethality, indicating its vital role in growth and development^11^. However, although it has been demonstrated to be significantly increased in multiple cancer types^12–14^, the exact role is poorly described.

NF-κB (Nuclear Factor K-light-chain-enhancer of activated B cells) transcription factors are critical regulators of differentiation, apoptosis, and immunity. Molecular defects promoting the constitutive activation of NF-κB pathway can support most steps involved in cancer transformation, for example, inhibition of cell differentiation and apoptosis, promotion of cell proliferation, angiogenesis, metastatic potential, and resistance to treatments^15^. And in this study, we aim to investigate whether H19 could be detected in bone marrow and used as biomarkers for MM, and define the NF-κB-related mechanism responsible for its cancer-promotive function.

## Materials and Methods

### Tissue samples

Bone marrow and peripheral blood samples were collected from thirty patients with MM (17 male and 13 female) who were hospitalized at Department of Hematology, the First Affiliated Hospital of Jinzhou Medical University from 2012 to 2016. For the use of clinical materials for research purposes, prior approval was obtained from the Medical Ethics Committee of Jinzhou Medical University (#LJMU 2016JZ-05217). The study was conducted according to the principles expressed in the Declaration of Helsinki. All samples were collected and analyzed with prior written, informed consent of the patients. The study was carried out in accordance with the institutional ethical guidelines and the use of human bone marrow tissues was approved by the Medical Ethics Committee of Jinzhou Medical University (#LJMU 2016JZ-05217). CD138 positive plasma cells were sorted, freshly frozen in liquid nitrogen, and then stored at −80 °C for further use. Bone marrow samples from 12 patients with monoclonal gammopathies of un-determined significance (MGUS), 10 patients with smoldering myeloma (SMM), and three healthy volunteers (non-tumor patients who received femoral heads excision, matched by gender and age) were collected as controls.

### Cell treatment

Human MM cell lines including OPM-2, U266, KM3, XG1, JJN3, RPMI, U1996, H929, and MM1S were obtained from American Type Culture Collection. All the cells were routinely maintained in RPMI-1640 medium supplemented with 10% fetal bovine serum (FBS) in a 37 °C humidified atmosphere of 5% CO_2_. Downregulation of H19 in cells was obtained by transfection with shRNAs. Two specific siRNA sequences were designed. Forward1, 5′-GCGGGUCUGUUUCUUUACUUU-3′; Reverse1, 5′-AGUAAAGAAACAGACCCGCUU-3′; and Forward2, 5′-TTCAAAGCCTCCACGACTCT-3′; Reverse2, 5′-: GCTCACACTCACGCACACTC-3′. The siRNA sequence and the control sequence (for scramble control shRNA: 5′-UUCUCCGAACGUGUCACGUTT-3′) were separately cloned into the GV102 vector by GenePharma Inc. Cells were plated in 6-well plates and transfected using Lipofectamine2000. After 24 h, the stably transfected cells were selected with 800 mg/mL G418 for at least one month. After selection and isolation of stably transfected clones, the clones were analyzed for H19 expression using quantitative real-time PCR.

PDTC, TNF-α and bortezomib were purchased from Sigma-Aldrich Inc. (St. Louis, MO), R&D Systems (Minneapolis, MN) and ActiveBiochem (Maplewood, NJ) respectively, and incubated with cells for 50 μM, 100 ng/mL and 10 nM respectively for 24 h.

### RNA extraction and quantitative real-time PCR analysis

Total RNA was extracted from tissues or cells using Trizol reagent according to the manufactures’ instructions. cDNA was synthesized from RNA using the reverse transcription Kit (cDNA first strand synthesis kit, Takara, Dalian, China). Real-time PCR was carried out using SYBR green PCR master mix (Applied Biosystems, Carlsbad, CA) to detect the expression of H19, and data were processed using 2^−ΔΔ^CT method and normalized to GAPDH^16^. The primer sequences used were as follows: GAPDH, forward: 5′-GTCAACGGATTTGGTCTGTATT-3′ and reverse: 5′-AGTCTTCTGGGTGGCAGTGAT-3′; H19, forward: 5′-TTCAAAGCCTCCACGACTCT-3′ and reverse: 5′-: GCTCACACTCACGCACACTC-3′.

### Protein extraction and western blot analysis

Cells were lysed in RIPA buffer (50 mM Tris/HCl, pH 8.0, 250 mM NaCl, 1% NP40, 0.5% [w/v] sodium deoxycholate, 0.1% sodium dodecylsulfate) with protease inhibitor and phosphatase inhibitor cocktail (Roche, Indianapolis, IN). Lysates were centrifuged at 20,000 g for 30 min at 4 °C. Lysate for nuclear P65 detection was prepared with a DNA/RNA/Protein Isolation kit (R6734-02, Omega, Norcross, GA) and the nuclear protein was extracted using a Nucleoprotein and Cytoplasm Protein Extract Kit (Beyotime, Shanghai, China) following with manufacturer’s instructions. Protein was subjected to a 10% SDS-acrylamide gel, transferred onto PVDF membrane and blotted using indicated primary antibodies overnight. Signals from HRP-conjugated secondary antibodies were generated by ECL Substrates (Beyotime, Shanghai, China). GAPDH was used as an endogenous control for normalization. GAPDH primary antibody (1:500) and histone antibody (1:500) was purchased from Santa Cruz Biotechnology, Inc. P65, phosphor-P65 and IκBα antibodies (1:1000) were purchased from Cell Signaling Technology Inc.

### Proliferation assay

Cell proliferation rates were evaluated using a CCK-8 assay. Cells were seeded in a 96-well culture plate at a density of 10,000 cells. CCK-8 reagent (10 μL) was added to 90 μL of the culture medium in each well. The cells were incubated subsequently for 1 h, and OD450 nm value in each well was determined by a microplate reader^17^.

### Trypan Blue exclusion viable cell assay

Cells were re-suspended in equal volumes of medium and stained with 0.4% trypan blue solution, and then counted using a haemocytometer. Viable cells number was assessed based on exclusion of trypan blue dye and cells that took up trypan blue were counted as dead cells.

### Soft agar colony formation assay

Cells were diluted in RPMI-1640 medium supplemented with 20% FBS, mixed with 3% low-melting point agarose solution, and plated on 6-well plates (1000 cells for each well). After cultured for 10 days, the colonies were stained with 0.1% crystal violet solution containing 80% methanol for 5 min. The number of colonies defined as >50 cells/colony were counted.

### Co-cultures of MM cells and hBMSCs

Primary bone marrow stromal cells from controls (hBMSCs) were isolated according to the method reported by Garayoa *et al*.^18^. Briefly, mononuclear cells from bone marrow aspirates were obtained by Ficoll-Paque density gradient centrifugation and cultured in DMEM media for 3–4 days. Non-adherent cells were removed, and stromal cells were selected. Co-culture experiments were evaluated using transwell inserts with 0.4 μm pores. MM1S or RPMI cells were seeded in the upper inserts at a density of 2 × 10^6^/mL and confluent hBMSCs was growing on the matched lower chamber. After 48 hours of incubation at 37 °C, the levels of IL-6 in the supernatants were measured using ELISA.

### Enzyme-linked immunosorbant assay (ELISA)

Concentrations of IL-8 and IL-6 in the cell culture supernatant and plasma samples were determined by sandwich-type ELISA, performed according to the manufacturer’s instructions (Boster, Wuhan, China). Absorbance was read at 450 nm, and the concentration was determined by comparing their optical densities to a standard curve.

### Statistical analysis

All data from 3 independent experiments were expressed as mean ± SD and processed using the SPSS 18.0 software. A P-value of <0.05 was considered to indicate a statistically significant result. The differences among the groups were estimated by Student’s t-test or one-way ANOVA. Non-parametric Kruskal-Wallis test and one-sample t-test were used to compare the expressions of H19 in bone marrows and in cell lines respectively. The Mann-Whitney U test and Spearman’s correlation analyses were used to analyze the relationship between H19 expression and IL-8 or IL-6 expression. Survival curve was estimated using a Kaplan-Meier analysis and compared using the stratified log-rank test (high level of H19 was defined as more than 16, which was the mean level of H19 in all the MM patients).

## Results

### H19 is upregulated in MM tissues and cells, and associated with clinical stages

**Figure 1:**
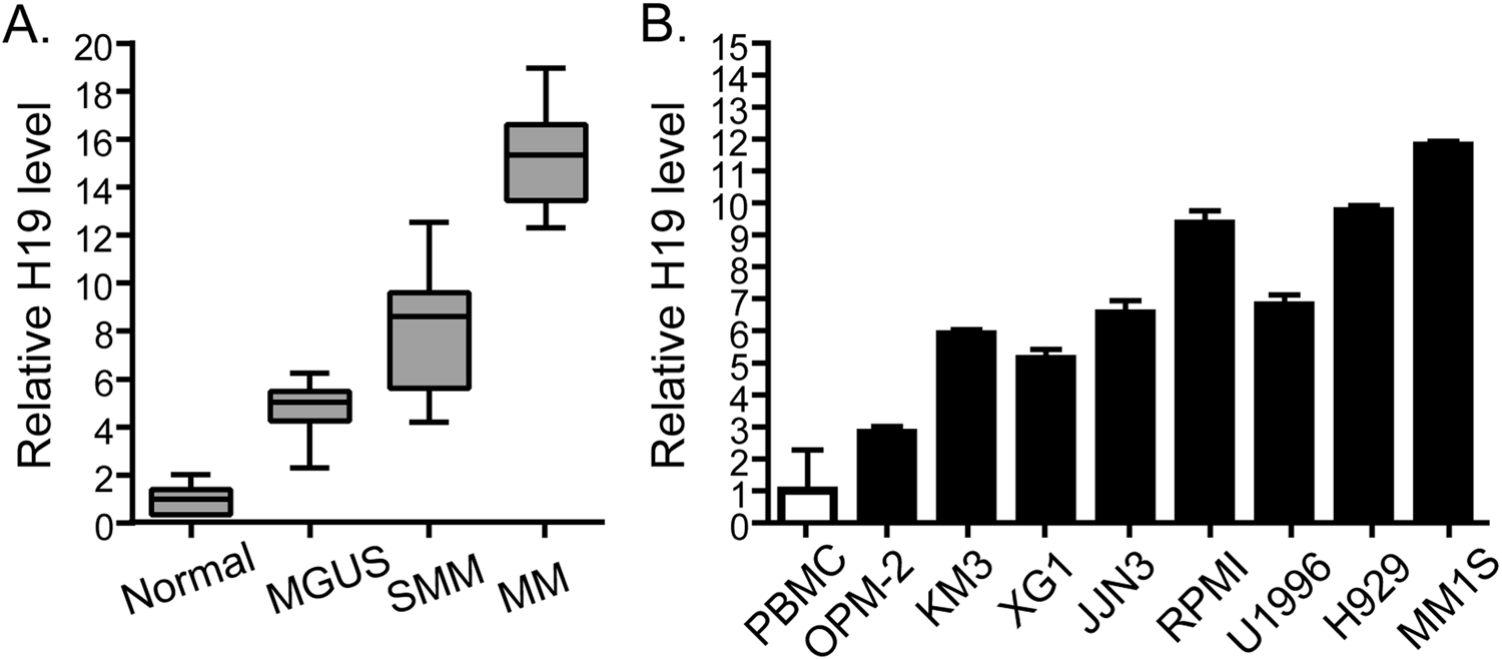
Increased expression of H19 in both MM bone marrow and cell lines. (**A**) H19 expression in bone marrow of MM patients compared with patients with MGUS, SMM and normal controls (three non-tumor patients who received femoral heads excision and were matched by gender and age). (**B**) H19 expression in nine MM cell lines compared with normal control peripheral blood mononuclear cells (PBMC) pooled from 3 healthy individuals. All the data were processed using 2^−ΔΔ^CT method and normalized to GAPDH expression.

We first tested the expression of H19 by real-time PCR in our collection of sorted CD138^+^ bone marrow biopsies from healthy controls, MM patients and subjects with the premalignant condition MGUS and SMM. The result revealed that compared to their age/gender-matched normal controls, H19 was significantly upregulated in bone marrow from patients with MM and to a lesser extent in samples from MGUS or SMM subjects (Fig. 1A, P < 0.001). Then we determined H19 expressions in nine human MM cell lines. All the MM cell lines showed a notable higher level of H19 compared to the normal control peripheral blood mononuclear cells (PBMC) pooled from 3 healthy individuals (Fig. 1B, P < 0.01). These data suggest that H19 is positively correlated with MM disease activity, and might promote MM progression.

### Knockdown of H19 inhibits MM cell growth

**Figure 2:**
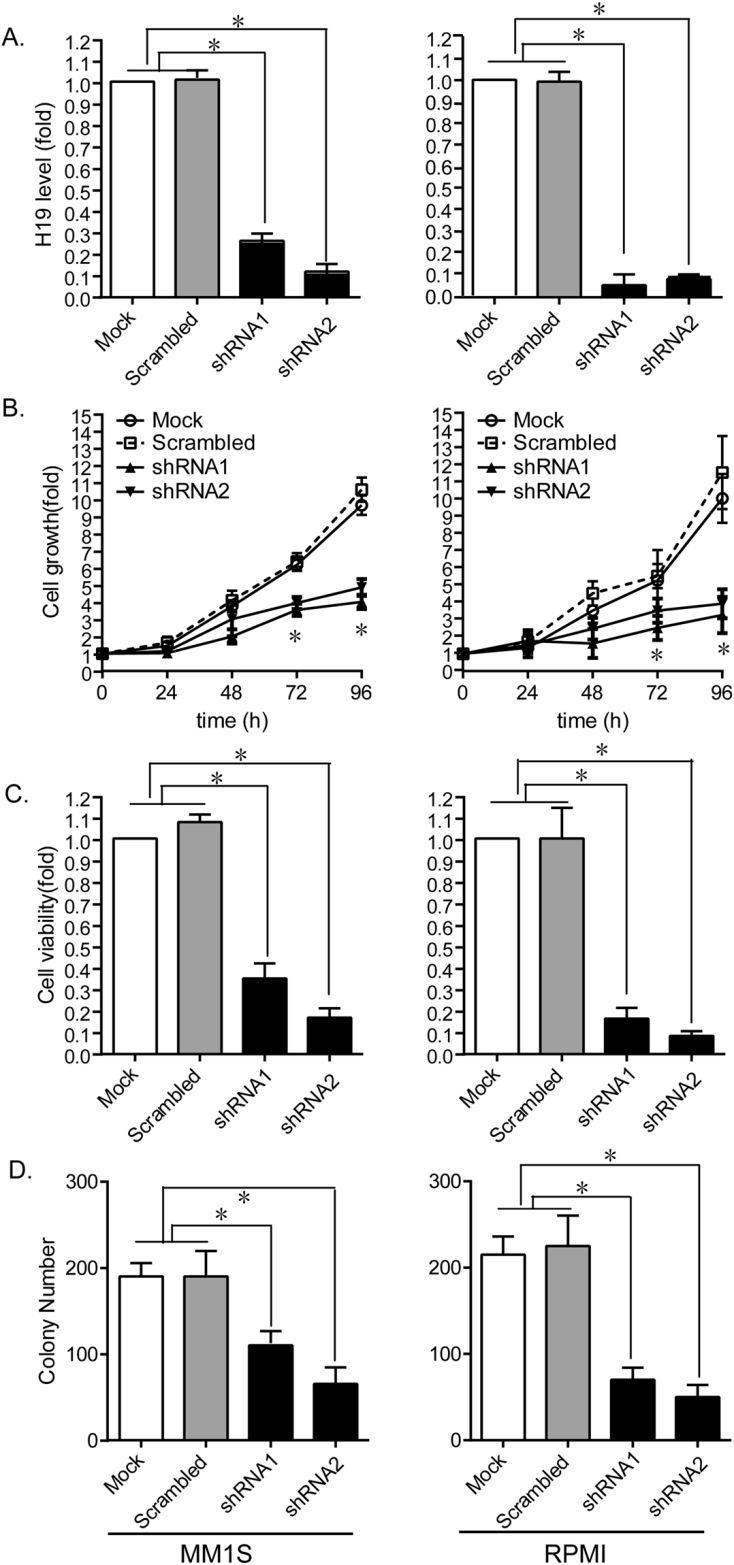
Effects of H19 on growth of MM cells. (**A**) Expression of H19 was significantly silenced by transfecting MM1S or RPMI cells with shRNA. Loss of H19 expression significantly inhibited cell (**B**) proliferation, (**C**) viability, and (**D**) colony formation. (*P < 0.01, Figure is representative of 3 experiments with similar results).

MM1S and RPMI cells presented with respectively higher expression of H19, thus we knocked down the expression of H19 by stable transfection with shRNA in these two cell lines and determine the effects of H19 on cellular biological function. As confirmed by PCR analysis, sh-H19 treatment significantly silenced expression of H19 (Fig. 2A, left MMIS, right RPMI). shRNA transfectants exhibited significantly decreased cell growth compared with either NC shRNA transfectants (Scrambled) or parental cells (Mock), determined by CCK-8 proliferation assay and Trypan Blue exclusion viable cell assay (P < 0.01, Fig. 2B and C). Colony formation assay showed similar results, and sh-H19 treated cells formed notably fewer colonies than controls (P < 0.01, Fig. 2D).

### Knockdown of H19 inactivates NF-κB pathway and suppresses cytokine secretion

**Figure 3:**
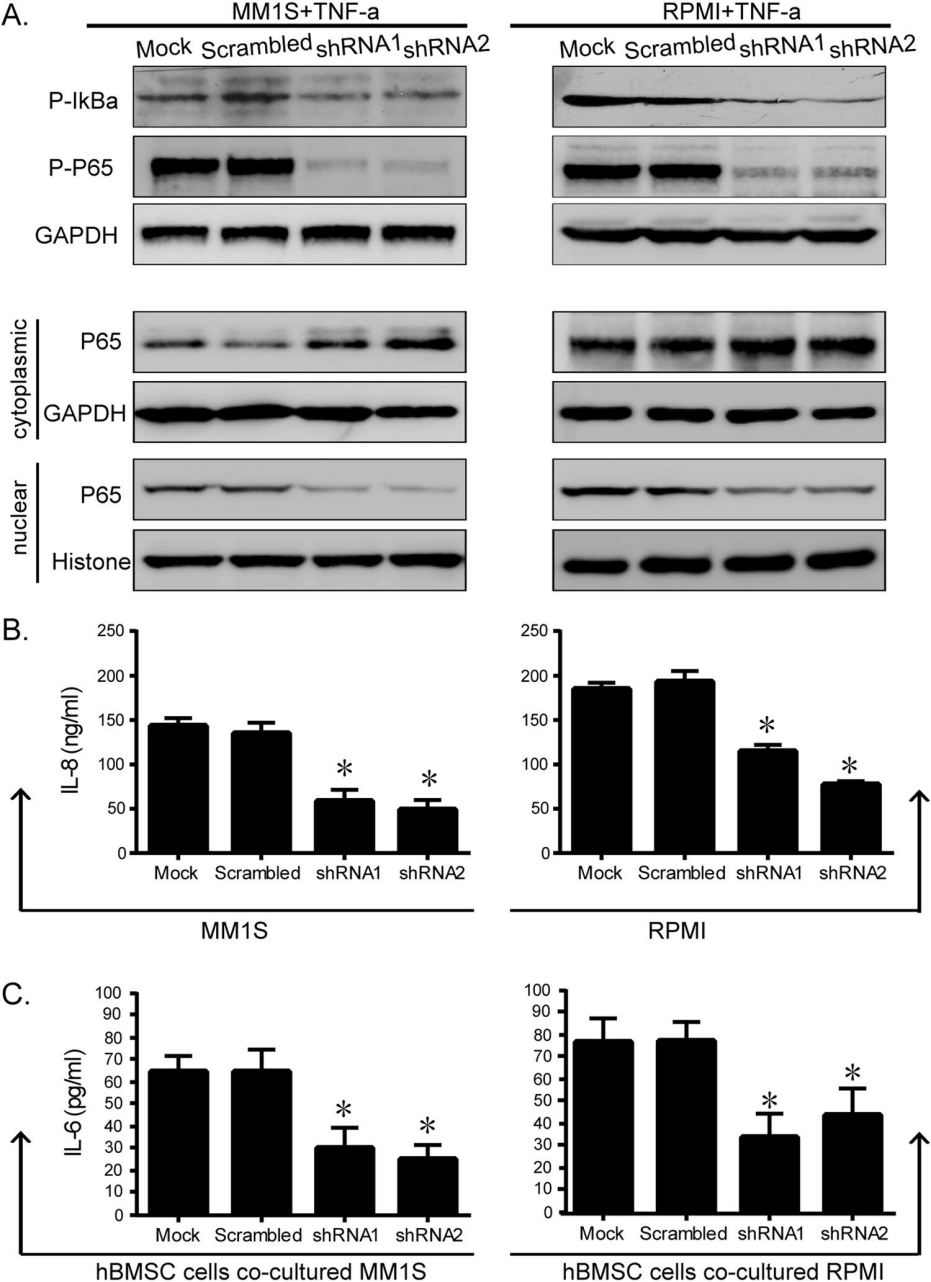
The activities of NF-κB and secretion of IL-8 were significantly repressed in H19 silenced MM cells. Secretions of IL-6 by hBMSCs were significantly decreased when co-cultured with H19 silenced MM cells. (**A**) Phosphorylated IκBα and P65, as well as cytosol and nucleous protein levels of P65 in H19 silenced MM cells were detected by western blot. GAPDH expression was used to normalize for equal loading. TNF-α (100 mg/mL for 24 h) was used to induce NF-κB activation. Secretions of IL-8 by MM cells (**B**) and IL-6 by hBMSCs co-cultured with MM cells (**C**) were detected by ELISA. (*P < 0.01, Figure is representative of 3 experiments with similar results).

The NF-κB family of transcription factors play an essential role for the regulation of malignant transformation, thus we tested the activation of NF-κB pathway to explore the potential underlying mechanisms of H19-related changes of cell growth. The phosphorylation of IκBα and the transfer of P65 from the cytoplasm to the nucleus were the premise of NF-κB activation. So western blot was performed to detect IκBα phosphorylation and P65 phosphorylation and nuclear accumulation. TNF-α (100 mg/mL for 24 h) was used to induce NF-κB activation. The results showed that neither IκBα nor P65 was activated in shRNA transfected cells in response to the TNF-α treatment as in the scramble control cells. TNF-α treatment could enhance the expressions of p-IκBα and nuclear P65, whereas H19 knockdown reduced these expressions (Fig. 3A, middle and lower level). Consistently, secretion of cytokine IL-8, also target of NF-κB, was suppressed by sh-H19 (Fig. 3B). Moreover, knockdown of H19 in MM1S and RPMI cells inhibited IL-6 secretion by hBMSCs after 48 hours of co-culture (Fig. 3C). Taken together, these data strongly indicated that H19 could regulate NF-κB signaling activation. This influence maybe contribute to the inhibitory effects of H19 on cell growth.

### H19 regulated MM cell growth synergistically with NF-κB pathway

**Figure 4:**
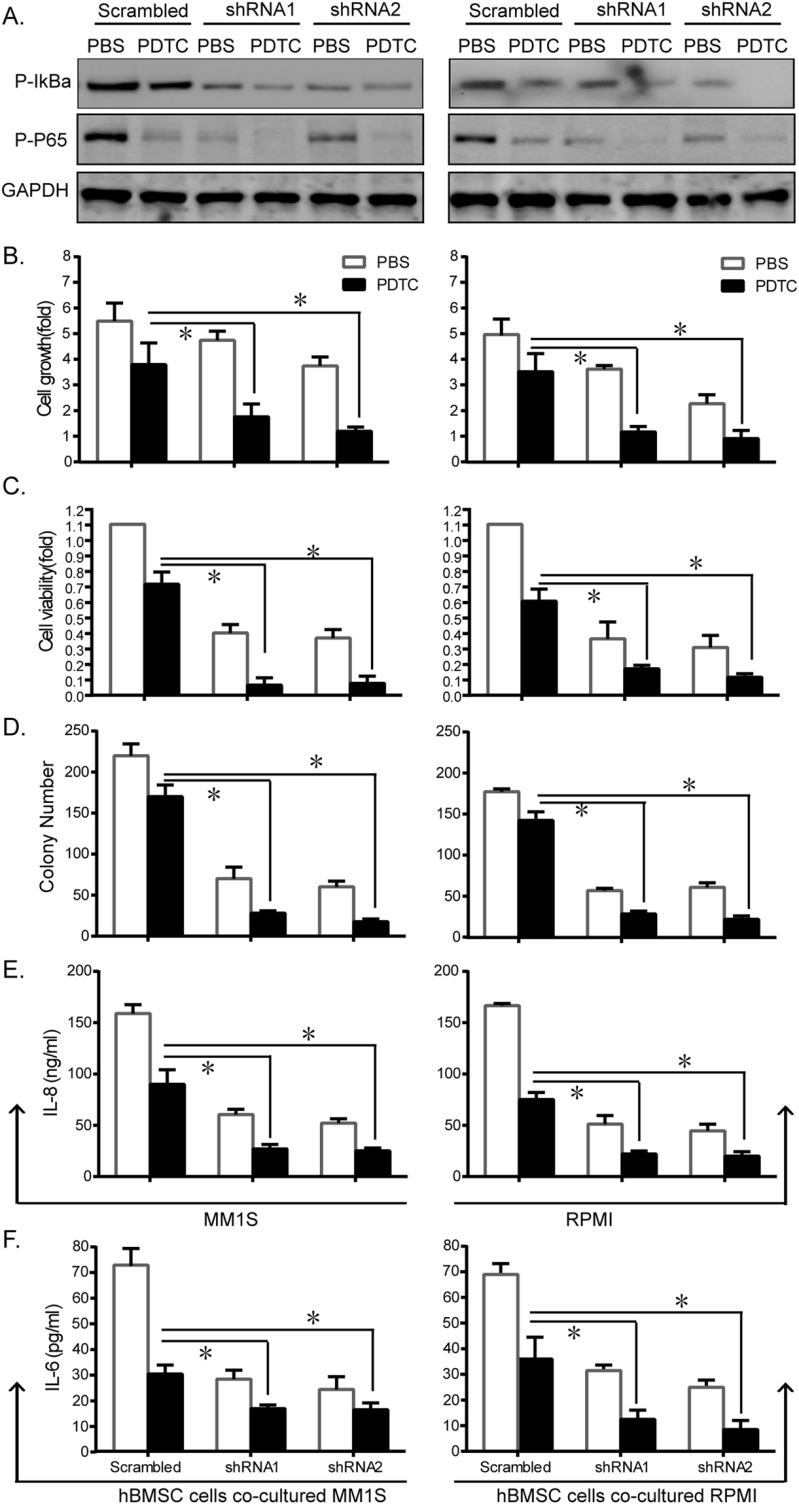
Inactivation of NF-κB participated in H19-regulated cell growth. Sh-H19 transfected MM cells were treated with NF-κB inhibitor PDTC (50 μM for 24 h) compared with treatment with PBS as negative control. (**A**) Western blot was performed to determine phosphorylation changes of IκBα and P65. Cell proliferation (**B**), viability (**C**), and (**D**) colony formation decreased significantly upon PDTC stimulation. The synergistic inhibitory effects were induced in terms of IL-8 secretion by MM cells (**E**) and IL-6 secretion by hBMSC cells (**F**). (*P < 0.01, Figure is representative of 3 experiments with similar results).

We treated shRNA transfectants with specific pharmacological inhibitor PDTC to investigate the effects of NF-κB on H19-related cell growth. As shown in Fig. 4A, the indicated inhibitor (50 μM) reduced the expressions of p-IκBα and p-P65. Consistently, cell proliferation decreased remarkably (Fig. 4B–D). To further investigate the interaction of H19 and NF-κB, we performed synergy experiment by treating H19-shRNA transfected cells with PDTC. Combination treatment induced synergistic inhibitory effects on cell proliferation (Fig. 4B), viability (Fig. 4C) and colony formation (Fig. 4D), compared to either shRNA or PDTC treatment alone. The synergistic inhibitory effects were also induced in terms of secretion of IL-8 by MM cells and IL-6 by hBMSC cells (Fig. 4E and F).

**Figure 5:**
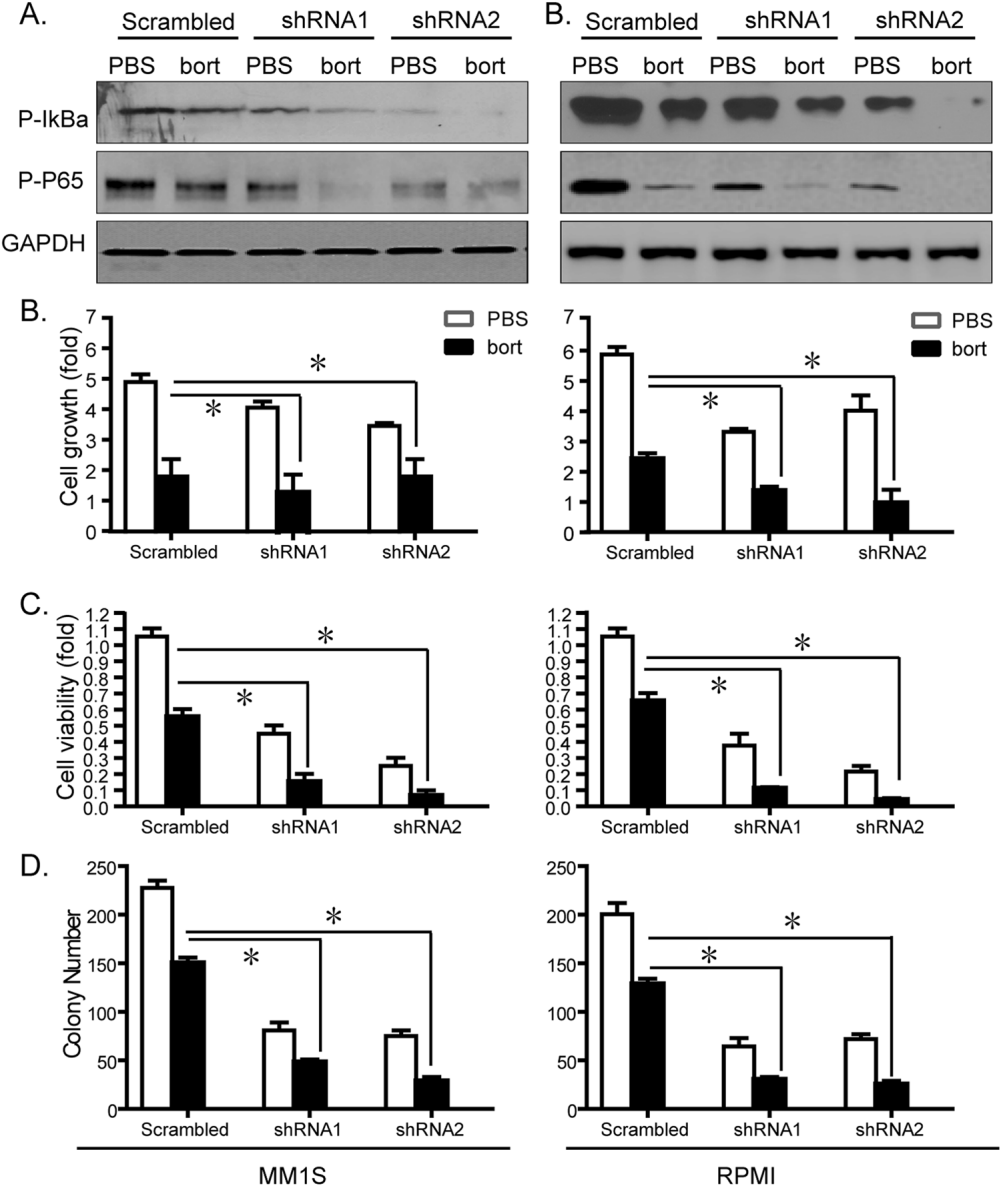
H19 depletion made MM cells more sensitive to bortezomib in terms of NF-κB activation and cell proliferation. Sh-H19 transfected MM cells were treated with bortezomib (bort, 10 nM, for 24 h) compared with treatment with PBS as negative control. (**A**) Western blot was performed to determine phosphorylation changes of IκBα and P65. Cell proliferation (**B**), viability (**C**), and (**D**) colony formation were determined respectively.

We further extended the above experiments with currently used anti-MM drug bortezomib. As shown in Fig. 5, H19 depletion also made MM cells more vulnerable to NF-κB (Fig. 5A) when treated by bortezomib. And sh-H19 cells were more sensitive to bortezomib in terms of proliferation and survival (Fig. 5B–D) too. Taken together, these results indicated that H19 functions as a potent oncogene through NF-κB pathway.

### Correlation of expression between H19 and cytokines

**Figure 6:**
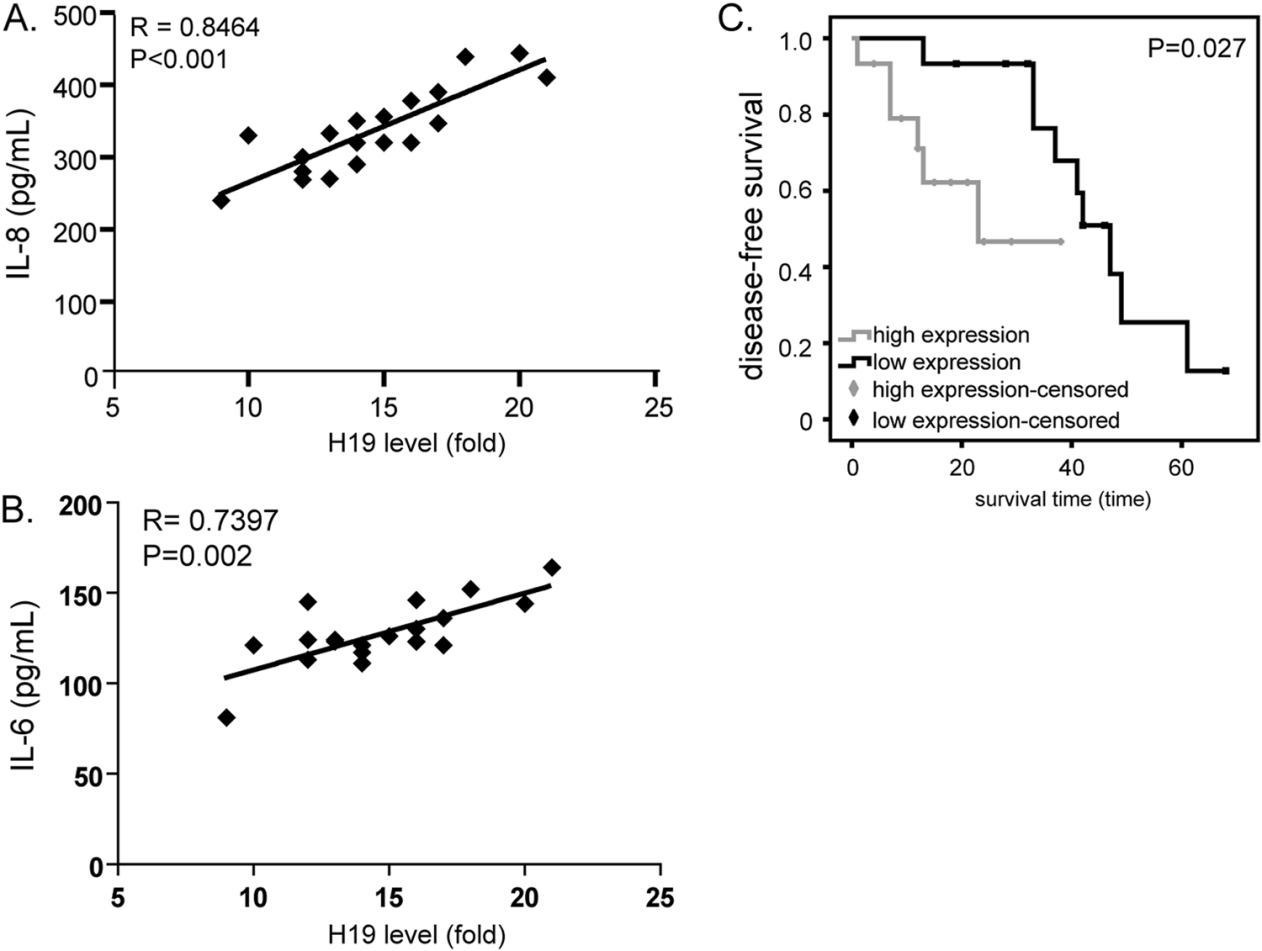
Correlation between bone marrow expression of H19 and circulating expression of IL-8 and IL-6 in MM patients. Survival analysis for MM patients. (**A**) Positive correlation between bone marrow expression of H19 and circulating expression of IL-8 in MM patients. (**B**) Positive correlation between bone marrow expression of H19 and circulating expression of IL-6 in MM patients. (**C**) Kaplan-Meier survival curves of MM patients relating to the status of H19 expression.

To confirm the relevance between H19 and cytokines, we investigated the bone marrow expressions of H19 and circulating IL-8 or IL-6 in MM patients. Relatively a large amount of IL-8 and IL-6 was secreted in peripheral blood in MM patients. Moreover, a positive correlation between H19 and IL-8 or IL-6 expression was observed (R = 0.8464, P < 0.001, Fig. 6A; and R = 0.7397, P = 0.002, Fig. 6B).

### Higher expression of H19 was associated with the poor prognosis of MM patients

To investigate the prognostic value of H19, the association of H19 with an disease free survival was evaluated using Kaplan-Meier survival curves with the log-rank test. The follow-up time ranged from 1 to 68 months. The median survival time of the group with higher expression of H19 was 23 months, and lower expression group was 47 months. The difference between the groups was significant (P < 0.01). The univariate survival analysis indicated that the survival rates of patients with higher expression of H19 was lower than patients with lower expression (P = 0.027, Fig. 6C).

## Discussion

**Figure 7:**
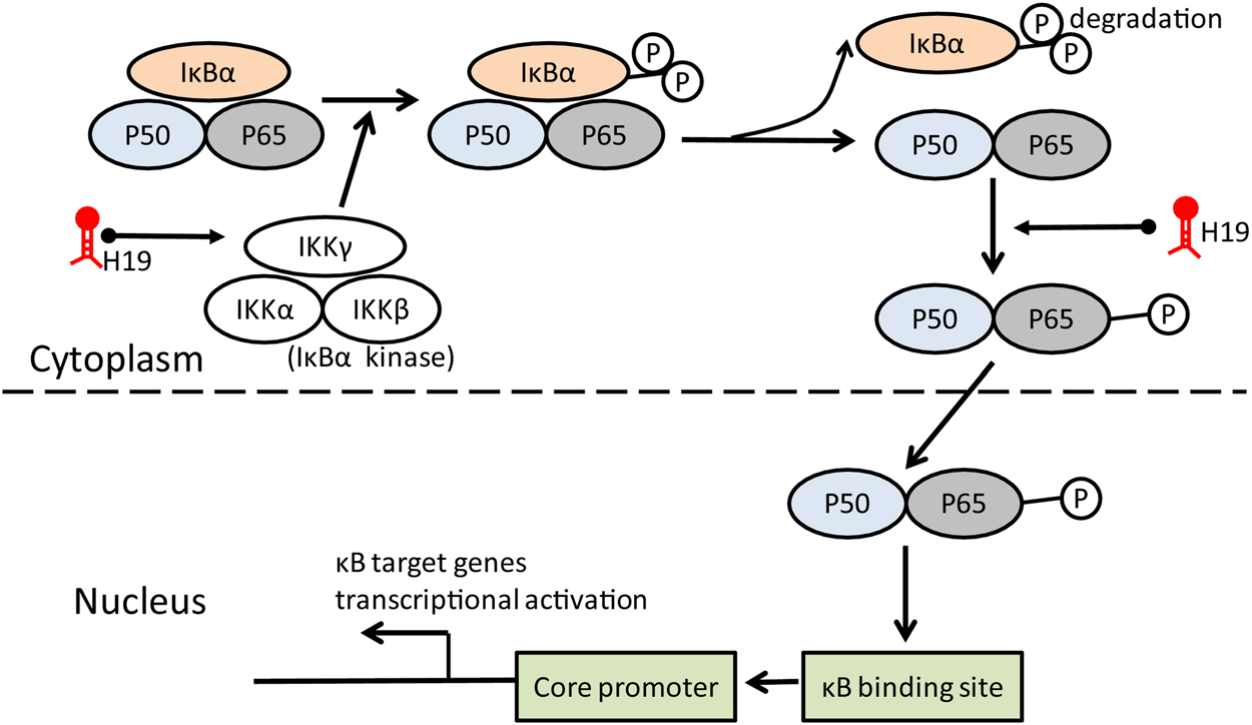
A schematic representation of the proposed model: possible mechanism of H19 via the NF-κB pathway regulated growth of MM. H19 induced phosphorylation of the typical IκBα, marking it for degradation, thus led to an enhanced nuclear NF-κB accumulation. Otherwise, H19 could directly promote P65 phosphorylation. Subsequently, nuclear P65 might bind to IL-6 and IL-8 promoter regions, and promoted their transcription.

Recently, increasing evidence has highlighted that lncRNAs are aberrantly expressed or mutated in human cancer, indicating that they may function as a novel class of oncogenes or tumor suppressor genes^5–9^. LncRNA H19 is highly expressed in fetus but decreased after birth. And it has been demonstrated in previous studies that H19 exerted two opposite effects depending on tumor types: it functioned as an oncogene in gallbladder cancer^19^, lung cancer^12,13^, esophageal cancer^13^ and ovarian cancer^20^; but exerted an opposite suppressive effects in hepatocellular carcinoma^21^, prostate cancer^22^ and Wilms’ tumor^23^. In our current study, we found that H19 functions as an oncogene in MM. H19 was highly expressed in human MM cell lines and bone marrow tissues. Knockdown of H19 could repress growth of tumor cells. Moreover, this inhibitory effect was related with inactivation of NF-κB pathway. H19 and NF-κB can regulate cell growth synergistically. Taken together, our finding provided a novel interpretation for the mechanism of H19-regulated cell growth in MM (Fig. 7).

As we mentioned above, studies reported different, even contradictory, roles of H19 in tumorigenesis in different tumor models, implying that the regulatory role of H19 may be specific to tumor context. In MM, H19 was commonly up-regulated in cultured cell lines and patient tissues, which is consistent with previous studies in gallbladder cancer^17^, lung cancer^12,13^, esophageal cancer^13^ and ovarian cancer^19^. H19 inhibition also induced simultaneous suppression of *in vitro* cell proliferation, viability, and colony formation of MM cells. However, studies about this mechanism are rare and limited.

The NF-κB pathway is a critical regulator in the progression and apoptosis of cancer cells including MM^24^. It is a family of transcription factors composed of structurally related proteins, including c-Rel, RelA (P65), RelB, NF-κB1 (P105 precursor of P50), and NF-κB2 (P100 precursor of P52). NF-κB activation is mediated by two signaling pathways, including the canonical or classical pathway and the non-canonical or alternative pathway. Upon cell activation, the typical IκBs (IκBα, IκBβ, IκBε) is phosphorylated by the IκB-kinase (IKK) complex in the cytoplasm. IκBα phosphorylation triggers the recruitment of the E3 ubiquitin ligase and the subsequent polyubiquitination of IκBα, marking it for degradation. This degradation releases the P65/P50 dimer that can translocate into the nucleus to activate the transcription of target genes^15^. Thus the P65/P50 dimer and IκBα complex are best understood as the main regulator of NF-κB activation^25^. Consistently, we for the first time demonstrated that H19 participated in cell growth in NF-κB-dependent way. Knockdown of H19 could downregulate pIκBα and nuclear P65, thus effect on the nuclear translocation of P65. However, further bioinformatics methods and chromation-immunoprecipitation analysis may be still needed to verify whether the promoter region of H19 contains a P65 binding site.

It is now well known that NF-κB is a key inflammatory pathway linking chronic inflammation and cancer transformation^26^. In the nucleus, activated NF-κB acts as a transcription activator and promotes the expression of a broad panel of inflammatory cytokines. Many studies have identified this. The activated NF-κB transcriptionally upregulates the expression of proinflammatory cytokines, such as IL-6, IL-1 and IL-2; chemokines, such as IL-8, MCP-1 and GRO-α, and growth factors such as VEGF and GM-CSF. These induced factors promote the proliferation of cancer cells, and also serve in angiogenesis, tumor invasion, and metastasis^27–31^. Similarly, we showed here that knockdown of H19 reduced secretion of IL-8 by MM cells and secretion of IL-6 by hBMSC cells. And bone marrow H19 level was positively associated with circulating IL-6 or IL-8 level in the same MM patients. In breast cancer, it was reported that NF-κB induced cancer progression through targeting IL-6 and IL-8^32,33^. Moreover, at the IL-6 promoter, the canonical NF-κB complex binds to the region close to the transcription start site and promotes IL-6 gene expression^34^. Although the precise molecular mechanism of H19-mediated regulation of expression of IL-6 and IL-8 in MM is still unknown, given the previous study, we speculated that H19 might function as a transcriptional promoter by binding to the promoter regions of these genes. And further studies are needed.

The NF-κB pathway is believed to be a potential therapeutic target for cancer^35^. For example, bortezomib, proteasome inhibitor which inhibits NF-κB activation, has been widely used to treat MM patients worldwide^36,37^. However, given NF-κB’s pleiotropic and ubiquitous functions, therapeutically targeting this pathway might be an insurmountable challenge. And lots of current NF-κB-targeting strategies indeed lack cancer cell specificity^38^. Our current study and previous publications have suggested that expression of H19 was cancer cell specific. More importantly, downregulation of H19 could inactivate NF-κB, and acts in synergy with NF-κB to inhibit cell growth in MM. As a result, downregulation of H19 expression could have an important implication for the clinical management of MM. Further investigation is needed, and whether H19 presents with any toxicity to normal cells should be clear up front.

In conclusion, we revealed the oncogenic effects of H19 in MM, and elucidated the potential mechanism by which H19 is implicated. Knockdown of H19 can suppress proliferation, growth and colony formation in MM cells by directly repressing NF-κB pathway. H19 expression in bone marrow is associated with poor prognosis, and inhibiting it represents a potential strategy against MM.
